# Analysis of Static Molecular Gradients in a High-Throughput Drug Screening Microfluidic Assay

**DOI:** 10.3390/molecules26216385

**Published:** 2021-10-22

**Authors:** Roman G. Szafran, Benita Wiatrak

**Affiliations:** 1 Department of Biochemistry, Molecular Biology and Biotechnology, Faculty of Chemistry, Wroclaw University of Science and Technology, ul. Norwida 4/6, 50-373 Wroclaw, Poland; 2 Department of Pharmacology, Faculty of Medicine, Wroclaw Medical University, Mikulicza-Radeckiego 2, 50-345 Wroclaw, Poland; benita.wiatrak@umed.wroc.pl

**Keywords:** HTS, lab-on-chip, cell culture, biochip, tumor-on-a-chip, microfluidics, diffusion, tumor microenvironment, drug discovery, microfluidic gradient generator

## Abstract

In this study, we thoroughly analyzed molecular gradient generation, its stability over time, and linearity in our high-throughput drug screening microfluidic assay (HTS). These parameters greatly affect the precision and accuracy of the device’s analytical protocol. As part of the research, we developed a mathematical model of dependence of the concentration profile on the initial concentrations of active substances in reservoirs and the number of tilts, as well as the dependence of the active substance concentration profiles in the culture chambers on the concentration profile of the reference dye in the indicator chamber. The mean concentration prediction error of the proposed equations ranged from 1.4% to 2.4% for the optimized parameters of the procedure and did not increase with the incubation time. The concentration profile linearity index, Pearson’s correlation coefficient reached −0.997 for 25 device tilts. The observed time stability of the profiles was very good. The mean difference between the concentration profile after 5 days of incubation and the baseline profile was only 7.0%. The newly created mathematical relationships became part of the new HTS biochip operating protocols, which are detailed in the article.

## 1. Introduction

A new drug development process is a long and expensive procedure that takes mean over 7 years (5.8–15.2 years) for a drug to travel from the research to the patient [1]. In 2020 O.J. Wouters et al. published a new survey on drug development costs [2]. They estimated from data for 63 therapeutic agents developed by 47 companies between 2009 and 2018, the median research and development investment (total, capitalized) required to bring a new drug to market be USD 985 million, and the mean was estimated to be USD 1336 million. For oncology drugs, those values were much higher and were estimated to be USD 2800 million and USD 4500 million, median and mean, respectively. Such huge expenses are motivated by the fact, that only one out of ten substances which after preliminary research begins preclinical tests reach the first phase of clinical tests, and subsequently only about 10% of them successfully pass all three clinical phases. One-third of costs are incurred during the preclinical stage on activity, drug-response, toxicity, ADME (absorption, distribution, metabolism, and elimination) tests. Despite this, over 50% of drugs that enter the clinical stage fail due to issues with efficacy. With this in mind, we can say that if we had better drug screening methods, we could eliminate ineffective substances before they reach the costly phases of clinical trials, saving billions of dollars a year and reducing development time for new drugs and vaccines. This is not insignificant given the effects of fast-spreading infectious pandemic diseases, such as SARS CoV-2, SARS, MERS-CoV, or Influenza. The approaching era of epidemic diseases forces us to intensify research into methods and devices for conducting rapid screening of medicinal substances and shortened procedures for their introduction to the market.

**Figure 1:**
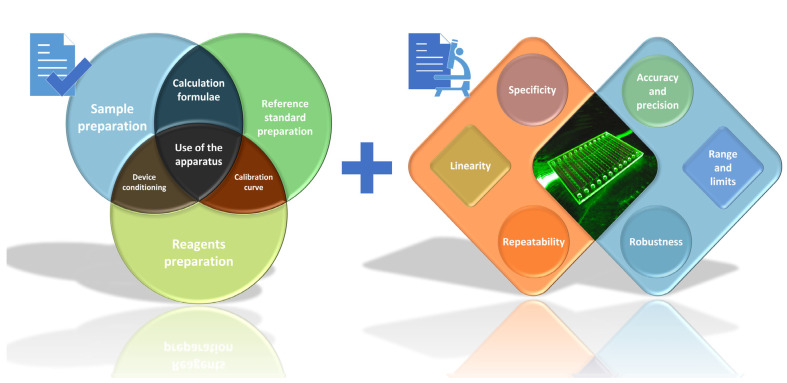
A microfluidic device-specific operation and industrial validation procedure elements.

Drug discovery requires trials on a large number of active substances and on a broad extent of their concentrations with high repeatability [3]. Figure 1 summarizes some of the important elements that were identified by the U.S. Food and Drug Administration (FDA) and must be determined in analytical procedures when used in drug discovery pipelines: the operation protocols (left), and the operation factors (right). The most important factors are: specificity, linearity, accuracy, specified range, precision (repeatability, reproducibility, robustness), detection and quantification limits, while the operation procedure characteristics should include: the sample preparation, preparation of reference standard and the reagents, use of the apparatus, generation of the calibration curve, use of the formulae for the calculation, and device preparation and conditioning [4,5]. In addition, automation and integration with a robotic analytical platform of high-throughput drug screening (HTS) microfluidic chips are needs that determine their successful market introduction [6]. Matching with standard laboratory equipment such as inverted fluorescent microscopes, multi-well plate readers, micropipette and incubators, and standardized cell culture and drug testing procedures are also crucial.

There are three different concepts of the microfluidic high-throughput drug assays existing in literature: (a) microfluidic chips with multiplexed cell culture chambers of microliter volume [7,8]; (b) microfluidic droplet cell culture chambers where each droplet’s content correspond to a reaction well [9,10], and (c) microfluidic chips with gradient cell culture chambers (CC) [11,12,13]. All of these concepts are interesting, nonetheless, since drug screening requires different drug concentrations to be tested for a dose-dependent cellular response, the integration of micromixers or gradient generators on the same chip creates a powerful tool that simplifies the screening procedure. Gradient generators provide an additional advantage—a controllable cell culture microenvironment with continuous linear, logarithmic, or Gaussian gradients of active substance [14]. Chemical gradients play an important role in mediating biological activity in vivo, including development, inflammation, cancer metastasis, drug delivery, embryogenesis, and wound healing. Cells respond to chemical signals in their environment by secreting signaling factors that either affect the secreting cell itself (autocrine) or affect other types of cells (paracrine). In many situations, e.g., in the case of cancer cells drug-resistance development, in vitro reconstruction of such signaling pathways and biomolecule gradients (e.g., growth factors, hormones, or cytokines) is crucial for proper drug-response trials [15]. The microfluidic devices with integrated gradient cell CC use two different approaches to generate gradients of substances—static mixers [16,17,18] (flow-based), and diffusive gradient generators (steady-state or diffusion systems) [19].

Static mixers that are based on the presence of the shearing which is caused by flowing streams are tree-shaped networks, altered tree-shaped networks, Y-shape junctions. The steady-state systems that are based on diffusion of species in stationary conditions are membrane systems, pressure balance systems, droplet-based methods [20]. Although flow-based gradient generators are the most popular, because the gradient is generated rapidly and its parameters can be controlled over time in long-term tests, in this configuration, autocrine/paracrine factors cannot accumulate in the cell’s environment because the streams of flowing fluid immediately carry away the secreted factors. Moreover, it was shown that cells that experience shear stress that does not occur in their natural environment respond differently to chemical gradients [21]. This configuration requires an external force or pressure gradient (pump) to trigger a flow that is difficult to precisely stabilize. Most diffusion-based gradient generators rely on gel-like materials [22], pressure balance between interconnecting chambers [23,24], or porous membranes [25] to create diffusive gradients, and suffer from long-term gradient formation or difficulties in stabilizing it. Pressure balance-based designs greatly improve the ability of diffusion generators to form dynamically controlled and stable gradients, but such generators still produce concentration gradients more slowly than convective generators. This drawback was partially solved by Jules VanDersarl and coworkers [26] by introducing a new microfluidic device of a hybrid architecture. In that device, the cell culture well is separated from a microfluidic channel located directly underneath the chamber by a nanoporous membrane. The chemical signals are transferred through the membrane into the large cell culture area, rather than propagate from the sides. The chemical gradient pattern generated by the tree-shaped gradient generator was transferred from the bottom channel to the cell culture area within 6 min. Although cells in this configuration are not subjected to shear stress, external pumps are still needed to generate dynamic gradients in the bottom channel, which appears to be a drawback for test automation.

Recently, we published a study where we introduced a three-chamber microfluidic device for cell culture in static gradients [27]. The device was used to analyze induced apoptosis of adenocarcinoma (LOVO) cells by three polyphenols: curcumin, trans-resveratrol, and wogonin. We found that polyphenols together with signaling factors released from cells into the microenvironment affect cell viability by stimulating cell apoptosis at lower polyphenol concentrations than in traditional multiwell cultures. Our research proved that the gradient microsystem is useful for routine laboratory testing and can better reproduce the tumor tissue microenvironment than standard multi-well cultures. Now we have developed its 12-chamber version intended for high-throughput drug screening. 

In this study, we will follow FDA recommendations to thoroughly analyze the molecular gradient generation procedure, its stability over time and linearity, which are the main parameters affecting the precision and accuracy of the analytical protocols of the invented microdevice. We will present the mathematical relationships linking the initial concentrations of active substances added to the reservoirs and the number of tilts of the microdevice with the distribution of concentration within the CC, as well as the relationship between the reference dye profile and the concentration profile of the active substance. 

## 2. Results and Discussion

### 2.1. High-Throughput Drug Screening Microdevice

**Figure 2:**
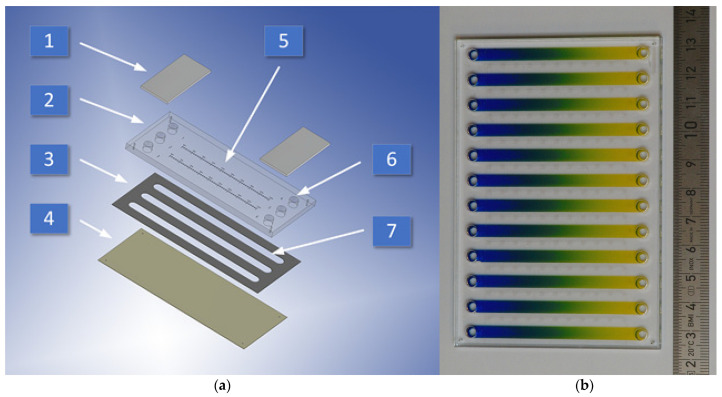
A microfluidic device for the high-throughput screening assay, (**a**) three-chamber configuration, (**b**) twelve-chamber configuration with developed counter-current molecular gradient of two dyes. 1—reservoirs cover, 2—top layer (lid), 3—middle layer (adhesive film), 4—bottom layer (base), 5—graduated scale, 6—fluid reservoirs, 7—culture chambers CC.

Two configurations of bio-chip were designed and fabricated: three (Figure 2a) and twelve-chamber (Figure 2b). The three-chamber device dimension is matched to the scale of microscope slide 76/31.2/3.3 mm (L/W/H)*,* while the twelve-chamber device 110/76/3.3 mm (L/W/H) perfectly fits our multi-well plate reader tray. Both microsystems were similarly made. All the device’s CCs are of the same dimension of 59/4.1/0.13 mm (L/W/H) and are terminated by cylindrical fluid reservoirs of the size of 3.3/3.8 mm (H/D). One chamber connected on both sides with reservoirs makes a channel of a total length of 66.6 mm. The transverse distance between centers of reservoirs fits the standard 96-well plate offset of wells—9 mm. The diameter of the reservoirs matches the standard diameter of the syringe tip. The millimeter-scale and dots along the chambers are engraved on the top surface of the top layer, making it easy to analyze the results and imaging them under the microscope. The CC, each of volume 34 μL, was made in the middle layer by cutting through the adhesive transfer film. All the chip construction materials were cut or engraved using a commercial CO_2_ laser system Versa Laser VLS 2.30 (Universal Laser System Inc., Scottsdale, AZ, USA) with a 30 W CO_2_ (wavelength 10.6 µm) pulsed laser source, a honeycomb flow-through cutting table, and a lateral gas-assist attachment.

In our biochip, the static gradients are generated quickly (in a few seconds) and repetitively by convective fluid movements caused by alternating tilting of the microdevice. The procedure of two-substances countercurrent gradients generation in a twelve-chamber microdevice is presented in the movie (Supplementary Material, Video S1). A detailed description of procedures of one-substance and two-substances countercurrent gradients generation is introduced in Section 4. The procedures of cell culture and their apoptosis evaluation were described previously [27]. Standard operation protocols assume that one of the CCs is filled with dye solution, we call it indicator chamber (IC), while the others are used for cell cultures in a gradient of the active substance or for blind trial. In the 3-chamber configuration (Figure 2a), actually, only one of three CCs is used for the test, while in 12-chamber microdevice (Figure 2b) ten CCs at once can be used for tests of the active substance. The reference protocols of microdevice operation are based on the use of a standard multi-well plate reader with a simple insert that allows the positioning of chips in the plate reader’s tray. Depending on the plate reader configuration, it allows measurement of absorbance, luminance or fluorescence along the chamber workspace in 10 (384-well configuration) or 20 points (1536-well configuration). We can say that one CC substitutes 10- or 20-well experiment (10 or 20 different concentrations), but actually it provides much more information, as the concentration gradient along the chamber is linear and continuous. In fact, therefore, the one-chamber experiment represents an infinite number of dilutions within a given in opposite reservoirs concentration range. Therefore, the accuracy of the obtained results is limited only by the resolution of the method of determining the test result and the stability of the gradient over time, but not on the dilution step of the active substance. In the 12-chamber high-throughput chip’s configuration, we can test at once 10 concentration ranges with an infinite number of dilutions of one or two substances in counter-current gradient conditions. One-chamber experiment needs only about 80 µL of tested substance solution. Additionally, as the volume/height of CC is very small, the concentration of signaling factors released from cells to their environment is high, so the 2D cell culture in microchannels better reflects conditions occurring in tissue cultures (3D) than surface multi-well cultures, while providing the same ease of analysis of the results. 

### 2.2. Surface Modifications of Cell Culture Chambers

In microfluidic cell culture systems for drug research, one of the major challenges is efficient, quantifiable, and reproducible immobilization of cells on a chamber’s surface for the purposes of exposure to the tested drug, analysis, or observation. This usually requires a special surface treatment of the channel to increase cell adhesion. The adhesion and proliferation of living cells depend on many surface properties, such as the surface charge, wettability, chemistry, microstructure, and surface roughness [28]. The surface modifications can be achieved by coating the surface with extracellular matrix (ECM) proteins such as collagen, fibronectin, laminin, or protein mixture such as Matrigel from Engelbreth-Holm-Swarm mouse sarcoma cells or synthetic PuraMatrix hydrogel. This mediates the specific interaction of cells to protein-coated surfaces via cell integrin receptors. The surface can also be modified by the cationic polylysine treatment. The presence of electric charges on a substrate surface affects the cell adhesion process as the vertebrate cells possess unevenly distributed negative surface charges on their membrane. Spatial variations of substrate surface properties allow microscale engineered cell co-cultures that are the crucial tool to create more in vivo-like cell culture models for drug research. 

The biochips, which we used in our study had modified CC surfaces. HTS microdevice has modified the channel’s surface for several reasons. First, to promote even distribution and firm adherence of cells to the bottom surface of the channel. Secondly, to increase the wettability of the channel’s surface to ease fluid penetration from reservoirs into the channel’s volume. A not-disturbed profile of fluid flow while filling the channels facilitates chip operation and prevents retention of air bubbles, that could be rally annoying phenomenon. Thirdly, we wanted to prevent the adsorption of color indicators and molecules of active substances on the channel’s surface. We tested several procedures and biological, chemical, and physical factors for channel modification, including: air low-temperature plasma, acrylic acid 10% (*v*/*v*) in water solution, (3-Aminopropyl)triethoxysilane (APTES) 10% (*v*/*v*) water solution, collagen (Roche cat. no. 11179179001), poly-D-lysine hydrobromide 0.01% (*w*/*w*) water solution, and hexadecyl trimethylammonium bromide (CTAB) 0.2% (*w*/*w*) water solution, all provided by Sigma-Aldrich. Finally, for simplicity and robustness, in the present work, we chose the microdevices with surface-modified chambers in low-temperature air-plasma (30 cm^3^/min air, 1 min, 30 W, 13.55 Mhz, 750 mTorr, PE-50 Plasma Eatch, Carson City, NV, USA) flowed by treatment by CTAB in water solution. The CTAB solution was introduced into the channels immediately after plasma treatment for 15 min at room temperature. Next, the solution was removed, and each channel was washed with deionized water (10 mL), dried by clean compressed air, and sterilized.

### 2.3. Analysis of the Light Absorbance Profile by a Chip’s Construction Materials

The linearity of an analytical procedure is its ability (within a given range) to obtain test results that are directly proportional to the concentration (amount) of analyte in the sample [4]. The analytical procedures that are combined with the biochip are exactly the same as used in standard biological test procedures and are based on spectroscopic detection of absorbance, luminescence or fluorescence of the sample. After cell staining, the results may be analyzed under epifluorescence microscopy or in the UV/VIS multi-well plate reader. Those methods are widely recognized, validated, and approved. Therefore, the main source of additional non-linearity in analytical procedures using a gradient chip may be chip design imperfections, e.g., the light transmittance inhomogeneity of the materials used to build the chip or the variation in CC height or layer thickness. Additionally, differences in concentration gradients between different CCs may be a source of non-linearity. In turn, a non-linearity of the generated gradient may lead to some variations of concentrations in time at the given point of CC, which also may lead to non-linear system response.

**Figure 3:**
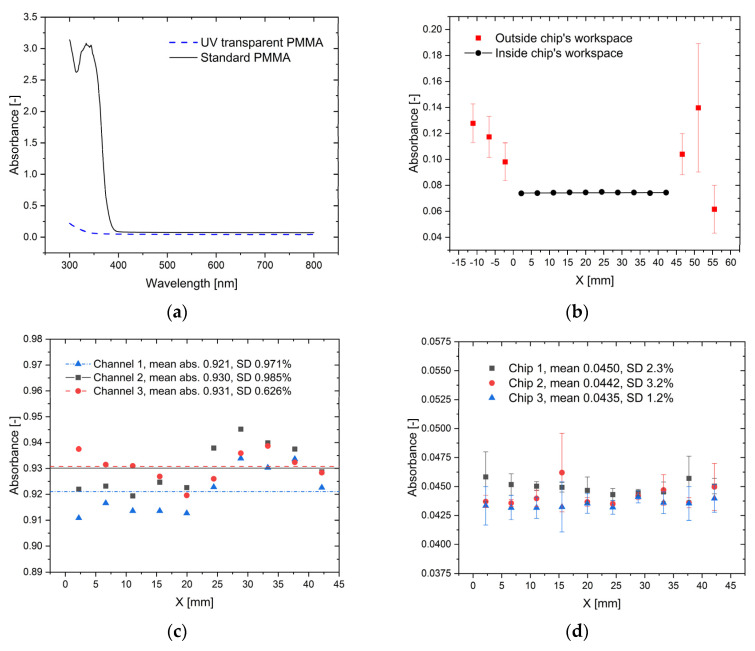
The light absorbance by the microdevice: (**a**) The spectrum 300–800 nm mean value for the empty CC; (**b**) The absorbance profile along of the empty CC at 668 nm, Plexiglas XT; (**c**) The absorbance profile along of CC filled with the indicator (MB 0.133%, CTAB 0.2%, 668 nm); (**d**) The absorbance profile along CC filed with 0.2% CTAB at 668 nm, Plexiglas XT.

In Figure 3, the results of the measurement of light absorbance in CC are presented. 

The microdevice is characterized by a low, constant value of background absorbance in the range of wavelength values 400–800 nm. We used two construction materials of the chip’s lid, UV-transparent PMMA (Plexigras GS) and standard PMMA (Plexigras XT) that have additives that absorb light with a wavelength shorter than 400 nm (Figure 3a). As was expected, the transmittance of Plexiglas GS is extended to 300–400 nm, which can be interesting for some analysis performed in the UV spectrum of light. We have to remember that usually excitation light in epifluorescence inverted microscopy illuminates the sample from the bottom, so the UV-blocking cover does not interfere with the imaging of the sample.

The mean absorbance along the CC in the chip’s workspace (668 nm, empty CC) was 0.0433 (SD 0.79%) for GS and 0.0744 (SD 0.71%) for XT (Figure 3b). The reservoirs’ covers made of the self-sticky sealing film are the source of large deviation of measured absorbance outside the chip’s workspace. Figure 3c shows the absorbance profile at 668 nm along the CC filled with 0.133% water solution of methylene blue. As the dye concentration is uniform in the channel, the observed divergence represents the variations in chamber height along the CC. As we can see, the standard deviation is below 1% and a similar dependence of absorbance on position for different channels of the same chip is observed. In Figure 3d, the absorbance distributions in CC filled with 0.2% CTAB water solutions (background) for three different chips (Plexiglas XT) are compared. The determined background was constant along the channel with the mean value 0.0442, SD 2.0%.

### 2.4. Gradients Evaluation

The relationship between the concentration of the indicator in IC and the concentration of the active substance in CC is based on the assumption that in all chambers the gradient distribution is exactly the same for each substance. It is true when all chambers are geometrically identical, fluids have the same properties and the protocol of gradient establishment was repeated precisely for each channel, so we can assume the identity of flow hydrodynamics. In practice, the procedure, in particular, is not sensitive to the angle and time of chip tilts, as fluids flow in chambers until reservoirs are emptied, and chambers’ surface wettability prevents fluid from escaping from chambers. The concentration gradient is not dependent on the molecular weights of substances as a mechanism of its generation is advective [29]. 

For one active substance, its concentration at a specific point in the chamber can be calculated from:

(1) $$\frac{C_{AMAX}-C_{AMIN}}{C_{IMAX}-C_{IMIN}}\cdot \;\left(C_{I\left(X\right)}-C_{IMIN}\right)\;+\;C_{AMIN}=C_{A\left(X\right)}$$

where *C_I_*_(*X*)_ is a dye concentration at a specific point, *C_IMAX_* and *C_IMIN_* are the maximal and minimal indicator concentrations in opposite reservoirs; *C_AMAX_* and *C_AMIN_* are the maximal and minimal concentrations of a substance in opposite reservoirs, and *C_A_*_(*X*)_ is the active substance concentration at a specific point. *C* can be expressed in any concentration unit.

For two counter-current gradients of substances, when the gradient of substance *A* have the same sense as the gradient of indicator, the concentration of *A* substance at a specific point in the chamber can be calculated from Equation (1), while the concentration of substance *B* (counter-current) at a specific point in the chamber can be calculated from:

(2) $$\left(1-\frac{C_{I\left(X\right)}-C_{IMIN}}{C_{IMAX}-C_{IMIN}}\right)\;\cdot \;\left(C_{BMAX}-C_{BMIN}\right)\;+\;C_{BMIN}=C_{B\left(X\right)}$$

With a well-defined gradient generation procedure, it is not necessary to measure the indicator concentration in the reference channel in a multi-well plate reader. While maintaining the repeatability of the procedure, the obtained concentration gradient should always be the same, so based on the indicator concentration profile once measured, the correct concentration of the test substance in the channel can be calculated each time.

### 2.5. Analysis of Linearity of Concentration Profiles

**Figure 4:**
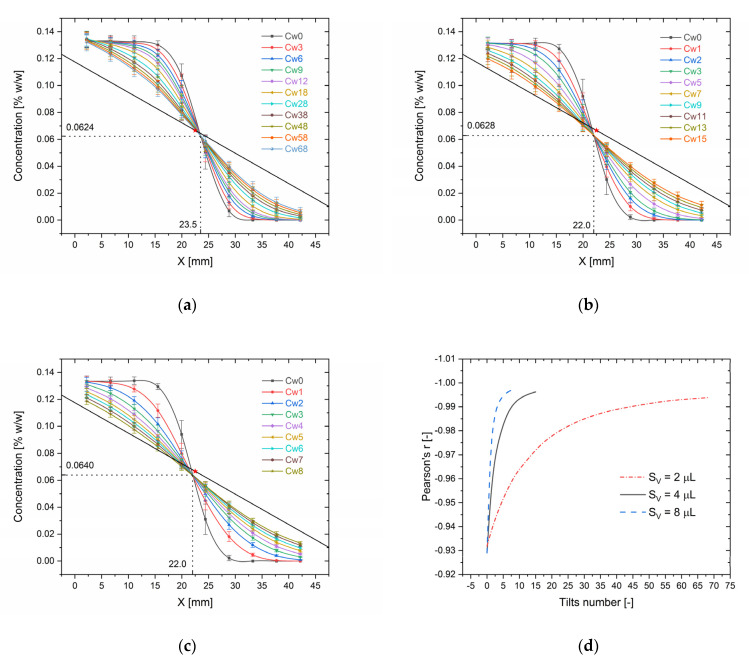
Analysis of influence of tilts number on gradient linearity *C_MAX_* = 0.1333, *C_MIN_* = 0 [% *w*/*w*]: (**a**) The distribution of the methylene blue concentration along CC for different numbers of tilts and (1.0 + 1.0) μL SV; (**b**) The distribution of dye concentration for (2.0 + 2.0) μL SV; (**c**) The distribution of dye concentration for (4.0 + 4.0) μL SV; (**d**) Dependence of Pearson’s correlation coefficient (*P_r_*) on number of device tilts (*T_N_*) for different SV. The straight line on Figures (**a**–**c**) with a red star dot (center mark) represents the theoretical dye concentration profile.

The shape of the concentration profile in CC strongly depends on the number of tilts (TN) of the microfluidic device. The greater the number of swings was performed, the closer to the linear function the substance gradient profile was reported (Figure 4a–c). 

Additionally, an increase in the swing volume (SV) of fluid added to the reservoirs reduced the number of swings needed to achieve a linear gradient profile. The linearity of profile is indicated by the value of Pearson’s correlation coefficient (Pr) (Figure 4d), that values rise above −0.99 for *S_v_* = 2 μL and *T_N_* = 48; *S_v_* = 4 μL and *T_N_* = 9 and *S_v_* = 8 μL and *T_N_* = 4, achieving −0.997 for 8 tilts and *S_v_* = 8 μL.

The straight line in Figure 4a–c represents the theoretical concentration profile according to the equation:

(3) $$C_{\left(X\right)}\left[\%\right]\;=\frac{C_{MIN}\left[\%\right]\;-\;C_{MAX}\left[\%\right]}{L\left[mm\right]}\cdot \;\left(X\left[mm\right]\;+\;X_{W}\left[mm\right]\right)\;+\;C_{MAX}\left[\%\right]$$

where *L =* 59 mm is the channel total length, *X_W_* = 7 mm is the distance from the channel’s beginning to the beginning of the chip’s workspace. The line connects two points representing the maximal and minimal concentrations at the opposite ends of the CC. The red dot star is placed in the middle of the line and represents the center of symmetry where in theory bunch of curves should intersect. Some small shifts of the curve intersection point are observed from the theoretical one. The mean errors were 3.0% and 5.4% for X and C, respectively. Using small SV causes a drastic increase in needed tilts, and results in an increase in errors caused by fluid evaporation from reservoirs during the gradient generation procedure. It also makes the procedure error-sensitive on inaccurate volumes dispensed by pipette. Although the profile linearity above *P_r_* = −0.99 is achieved for 4 and 9 tilts (8 and 4 SV), in practice, we suggest performing at least 15–25 tilts (left-right swing) with 4 μL of SV or 8–10 tilts with 8 μL of SV, especially when long-term stability of the profile over time is essential. In this case, according to Equation (3), the maximum and minimum concentrations of the tested substance added to the reservoirs should be approximately 10% above/below the maximum and minimum concentrations required for the tests. Otherwise, when the lowest possible deviation from the initial concentrations is desired, a small SV (1 + 1) with a large number of tilts (>30) should be used. 

**Figure 5:**
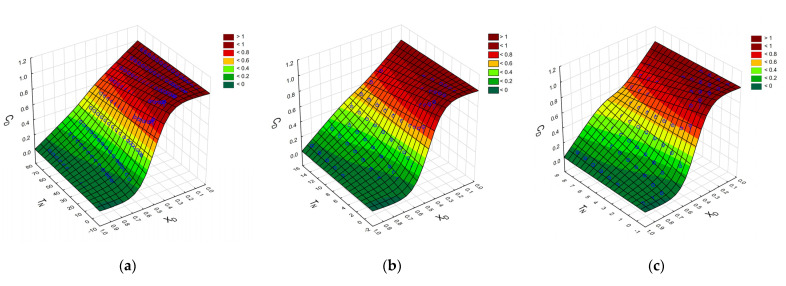
The surface plots of the function (4) approximating experimental data (blue circles) for: (**a**) S_V_ = 2 μL; (**b**) S_V_ = 4 μL; (**c**) S_V_ = 8 μL. Color bar scale for C_D_.

Based on the data presented in Figure 4, we propose an Equation (4) in the form of a generalized logistic function (Richard’s curve) that relates the normalized concentration of the substance $C_{D\left(X\right)}=\frac{C_{A\left(X\right)}}{C_{AMAX}}$ with a dimensionless distance from the CC’s beginning of workspace $X_{D}=\frac{X}{X_{MAX}}$ and the number of tilts *T_N_* for different dimensionless SV: $S_{D}=\frac{S_{V}}{V_{CC}}$, where *X_MAX_* is the length of the CC workspace, *C_AMAX_* is the maximal dye concentration in the reservoir and *V_CC_* is the total volume of CC.

(4) $$C_{D\left(X\right)}=A\cdot \;\left(T_{N}-X_{D}\cdot T_{N}\right)\;+\;\frac{1-A\cdot T_{N}}{1+exp\left(k\cdot \;\left(X_{D}-X_{0}\right)\right)}$$

where *X*_0_ is the value of the sigmoid’s midpoint and *k* is the logistic growth rate or steepness of the curve. *X*_0_ value ranges between 0 and 1, since *X_D_* has the same range of values due to normalization. The A parameter controls the rate of change of the shape of the curve (from sigmoid to straight line) with the increase in TN. In Figure 5, the surface plots of the function (4) approximating experimental data are presented. 

Table 1 summarizes parameters of Equation (4) experimentally determined for different values of SV.

Equation (4) with the relationships (1) and (2) allows the prediction of the concentration of tested compounds at any point of CC without the need for measurement of an indicator concentration gradient in the IC. However, this needs great caution and requires strict adherence to the gradient generation procedure described in Section 4 and good stability of the gradient profiles over time.

### 2.6. Analysis of the Mutual Relevance of Concentration Profiles

**Figure 6:**
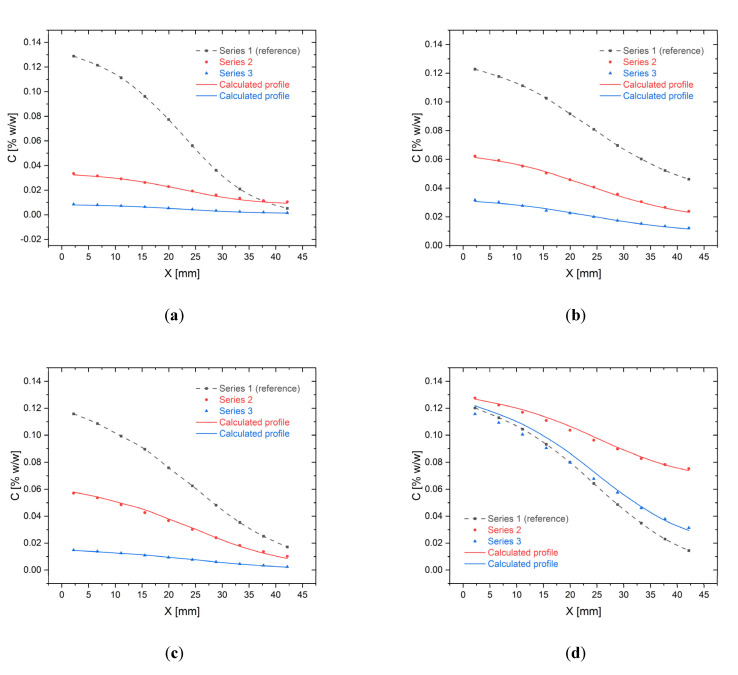
The relevance of concentration profiles-comparison between experimental data (points) and profiles calculated from Equation (1) (lines) for different dilution ranges and starting concentrations: (**a**) Profiles for 4 μL SV and 15 tilts; (**b**–**d**) Profiles for 4 μL SV and 25 tilts.

Figure 6 compares the experimentally determined dye concentrations along the CC with the profiles calculated from Equation (1). In each chip, three different profiles were prepared according to the procedure described in Section 4.

The concentration profile in the first channel was treated as a reference and was used to determine the other two profiles. In Figure 6a the reference concentration varies between 0.1333% and 0% (series 1), while the second profile was established between 3.33 × 10^−2^% and 8.333 × 10^−3^% (series 2), and third between 8.333 × 10^−3^% and 1.0416 × 10^−3^% (series 3), *S_V_* = 4 μL and *T_N_* = 15. The relative mean errors were: 4.3% (SD of error 4.2%) and 8.0% (SD of error 5.9%) for series 2 and 3, respectively. Figure 6b shows profiles established between 0.1333% and 0.0333% (reference, series 1), 0.0666% and 0.01666% (series 2) and 0.0333% and 0.008333% (Series 3), so each time the dilution was four times, *S_V_* = 4 μL and *T_N_* = 25. The relative mean errors were lower than in the first case: 1.4% (SD of error 0.94%) and 2.4% (SD of error 1.9%) for series 2 and 3, respectively. In turn, Figure 6c shows profiles established between concentrations 0% and 0.1333% (series 1), 0.0666% (series 2), 0.01666% (series 3), *S_V_* = 4 μL and *T_N_* = 25. The mean relative errors were: 4.4% (SD of error 4.5%) for series 2 and 3.4% (SD of error 3.6%) for series 3. The last Figure 6d shows profiles established between concentrations 0.1333% and 0% (series 1), 0.0666% (series 2), 0.01666% (series 3), *S_V_* = 4 μL and *T_N_* = 25. In this case, the mean relative errors were: 1.5% (SD of error 0.85%) for series 2 and 5.7% (SD of error 2.4%) for series 3.

A good match of the profiles was observed in all cases. We do not observe an increase or decrease in the compliance of the profiles with the change of the number of tilts, however, it seems that the best matching of the profiles was obtained for the same dilution range for the reference case and the analyzed case (Figure 6b). It should also be noted that in all cases the concentration ranges measured in the CC workspace differ from the concentration ranges of the mixtures added to the reservoirs. The increase or decrease in the initial concentrations at the ends of the chip’s workspace depends on the dilution range—the smaller the dilution range, the smaller the deviation from the starting concentrations.

### 2.7. Analysis of the Gradient Stability over Time

**Figure 7:**
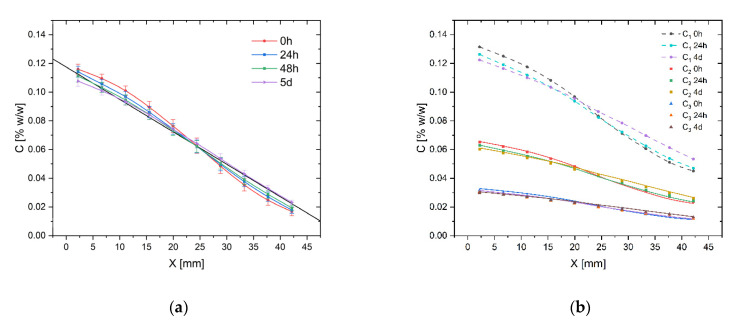
The gradient stability over time for 25 tilts and 4 μL SV; (**a**) A dye concentration profile for *C_MIN_* = 0% and *C_MAX_* = 0.1333%; (**b**) A dye concentration profiles for concentration ranges: C_1_ (0.1333–0.0333%), C_2_ (0.0666–0.01666%), C_3_ (0.0333–0.008333%). Dashed lines—reference profiles, solid lines—profiles calculated from Equation (1).

The dye concentration profiles in CC prepared according to the procedure described in Section 4 for 4 μL SV, 25 tilts, and different times of incubation are compared in Figure 7. Figure 7a shows the concentration profiles after 0, 24, 48 h, and 5 days of chip incubation for the maximum and minimum concentrations of dye added to the reservoirs: 0.1333% and 0%. The straight line in Figure 7a represents the theoretical concentration profile calculated from Equation (3). 

**Figure A1:**
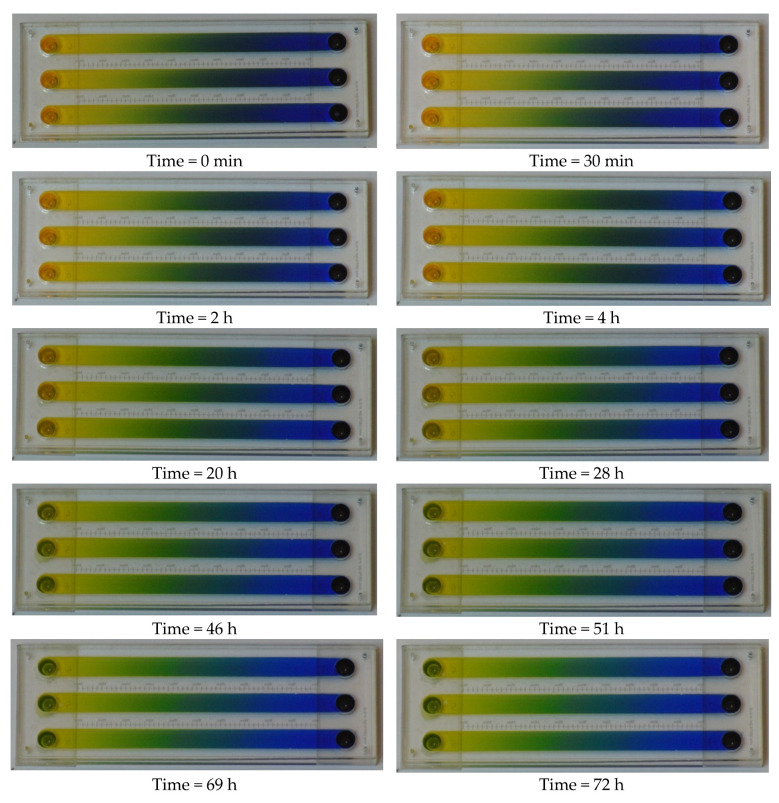
Visualization of stability of color indicators (fluorescein and methylene blue) counter-current concentration profiles over time.

As expected, due to molecular diffusion, the concentration profile becomes closer to a linear function over time. The Pearson’s correlation coefficient increases from −0.9960 to −0.9988 after 48 h and 5 days of incubation. The mean difference between an initial profile and measured profile after 24 h was 4.0%, 6.4% after 48 h, and 7.0% after 5 days of incubation. The concentration profile after 5 days becomes close to a straight line, and its changes are becoming the smallest and depend on the changes in the concentration of dyes in the reservoirs, which flatten the concentration profile in CC but also reduce the diffusive driving force and, consequently, slow down the equalization of concentrations. Additionally, the diffusive flux depends on the size and spatial structure of diffusing molecules, temperature, and the channel’s cross-section area. The stability over time of two counter-current dye concentrations profiles (fluorescein and methylene blue) in CC are visualized in Figure A1. The color of the solution in reservoirs slightly changes over time, while the concentration profile in CC is steady over 72 h. In Figure 7b, three concentration profiles after 24 h of incubation with the initial distributions of dye in CC are compared. In each case, the initial concentration was diluted four times along the CC and the C_1_ profile (0.1333–0.0333%) was treated as a reference for calculation from Equation (1) the other two (solid lines) profiles. Changes in the profiles over time even improve their mutual relevance. The mean errors of prediction for profiles C_2_ and C_3_ were 2.5% (SD of error 2.1%) and 5.0% (SD of error 2.0%) initially and decreased to 1.6% (SD of error 1.2%) and 3.3% (SD of error 1.5%) after 24 h. 

In all cases, we observed good stability of concentration profiles over time. Drug screening usually requires the active substance to be in contact with cells for several to several dozen hours, during which the medium in the reservoirs is changed at least once a day. The procedure of media exchange in reservoirs keeps the active substance concentrations in the reservoirs at a constant level, even for long-term cultures that exceed days or even weeks. The protocol of media exchange in reservoirs is described in Section 4. Changes in the dye profile over time reflect with high accuracy changes in the concentrations of the test substance along the CC when their molar masses are similar. 

## 3. Summary and Conclusions

In this study, we analyzed the molecular gradients generated in our microdevice intended for high-throughput drug screening. In particular, we analyzed gradients linearity and stability over time. We proposed Equations (1) and (2) which relate the indicator concentration with a concentration of the active substance in CC for one and two substance counter-current gradients, respectively. We also developed the relation (4) that links the dimensionless concentration of an active substance at a given point within CC with tilts number, initial concentrations of a substance in reservoirs and fluid swing volume. Equation (4) with the relationships (1) and (2) allows the prediction of concentration of tested compound at any point of CC without the need for measurement of an indicator concentration in the IC. The analysis of the mutual relevance of concentration profiles showed good agreement between the reference profile in the IC and the evaluated profile in the CC. The best agreement was obtained when the fold of the dilution was the same for the indicator and the assessed profile. In this case, the mean prediction error ranged from 1.4% to 2.4% and did not increase with the incubation time. The analyzed concentration profiles showed very good linearity and stability over time. For 25 tilts and SV = 4 μL, the Pearson’s correlation coefficient reached value −0.996. The profile linearity increases over incubation time, and the Pr value reaches −0.9988 after 5 days of incubation. The mean divergence between the initial profile and the profile measured after 24 h was 4.0%, 6.4% after 48 h, and 7.0% after 5 days of incubation. The biochip characterizes high concentration linearity and stability over time. The use of the reference dye concentration profile in determining the concentrations of the active substance leads to an increase in the robustness and precision of the analytical procedures. 

Comparing our microfluidic chip with other microfluidic gradient generators, its main advantage is its simple construction and operation, which is adapted to standard procedures and devices used to conduct cell cultures and methods of analysis of test results (inverse fluorescence microscopy, spectroscopic methods), without the need to use additional equipment such as pumps or valve systems, staff training, additional time consumption and costs generation. The fast-convective gradient generation and its excellent stability over time, as well as the high repeatability and reproducibility of gradients, distinguishes our solution from others. The original features of the device as well as verified detailed operating protocols and derived mathematical relationships allow the device to be used in routine tests in a repeatable and effective manner. The device’s features comply with the FDA recommendations for medical procedures and devices. The PDMS-free fabrication technology of HTS microdevice, which is based on laser ablation of construction materials, is easily scalable to mass production and hence gives the hence of rapid launching the product on the market.

The conclusions drawn from the experiments are consistent with the observations made previously during the practical tests of the device [27].

## 4. Materials and Methods

### 4.1. The Calibration Curve of Methylene Blue in Microdevice

**Figure 8:**
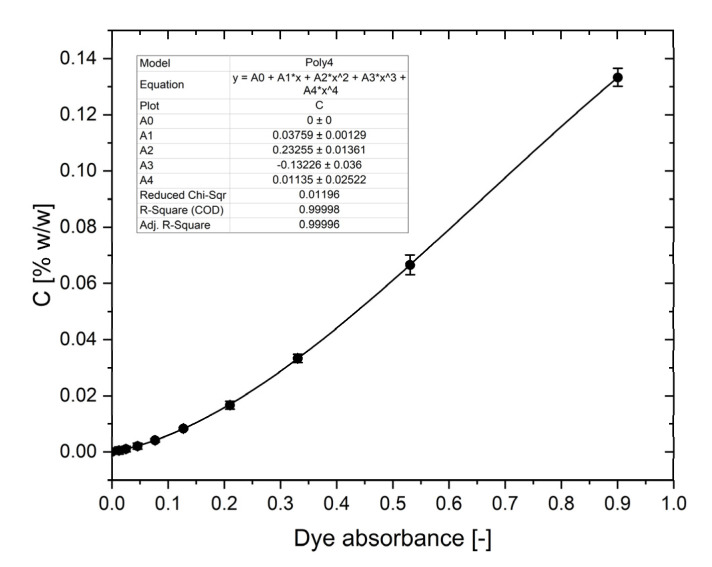
Calibration curve of MB in 0.2% CTAB water solution measured in microdevice’s CC at 25 °C. MB absorbance without background.

The microdevice with chambers filled in methylene blue (MB) solution in water with CTAB (0.2% *w*/*w*) was placed in a holder that fits a multi-well plate rider’s tray (384-well plate) and analyzed using a standard equipment procedure at the 668 nm wavelength (maximum absorbance of MB). All the reagents were provided by Merck, Darmstadt, Germany. The absorbance was measured along the IC within its workspace and the mean value for each dye concentration was determined. The calibration curve was prepared for several known concentrations of MB and their absorbance was measured in a microdevice at 25°C. The dependence of the dye concentration *C_I_* vs. absorbance *x* after subtracting the background absorbance was approximated by the polynomial equation, which determined parameters are presented in Figure 8.

In our investigations we used a modified version of the polynomial equation extended by the temperature factor:

(5) $$C_{I}=\;\left(A_{0}+A_{1}\cdot x+A_{2}\cdot x^{2}+A_{3}\cdot x^{3}+A_{4}\cdot x^{4}\right)\;\cdot \;F_{T}$$

where *A*_0_–*A*_4_ are experimentally determined parameters and *F_T_* is the temperature factor that considers changes of the dye absorbance with temperature. Its value was set to 1 for t = 25 °C, while *F_T_* > 1 for t < 25 °C, and *F_T_* < 1 for t > 25 °C. The correct value of the parameter was determined each time with the use of a reference system—a microdevice filled with a dye with a concentration of 0.1333% with CTAB (0.2% *w*/*w*), kept at the same temperature as the tested samples.

### 4.2. The Reference Protocols of Gradient Generation in the Culture Chambers

The procedure of generation of a concentration gradient of reactant (bio-active substance) is based on a convective, reciprocating fluid movement in chambers, that is induced by an alternating tilting of a chip. As a color indicator, a water solution of dye-methylene blue 0.133% (*w*/*w*) in CTAB water solution (0.2% *w*/*w*) was used, while a CTAB 0.2% (*w*/*w*) water solution was used as a complementary liquid added to the second reservoir of IC.

#### 4.2.1. One-Substance Gradient Generation

After filling the chambers with the base fluid (culture medium or CTAB (0.2% *w*/*w*) water solution), the 15 μL of the active substance solution in culture medium or in water CTAB solution of the maximal tested concentration was added to the inlet reservoirs of CC and the same volume of an indicator (dye) solution was added to the inlet reservoir of IC. The microsystem was raised at an angle of 60° to introduce the fluids into the chambers and held in that position until all the reservoirs are emptied. Then, fluids were removed from the outlet reservoirs and 2.0 μL (unless otherwise specified) of appropriate fluids were added to each reservoir (total volume of swing fluid was 4 μL). In the next step, the gradients were prepared by tilting alternately of the microsystem by 60° once by once (left and right), repeating the process 25 times (unless otherwise specified). The repetitive tilting of the microsystem induces the reversed-direction transient flow of fluids in CC and IC that is responsible for convective transport of solute in a culture chamber, which causes the gradient generation. Next, the excess liquid was removed from the reservoirs. The microdevice was inclined at an angle of 30° and the lower located reservoirs were filled with 25 μL of fresh liquid that corresponds to the adjacent chamber content (tested compound solution, indicator solution or complementary liquid, cell culture medium). The lower located reservoirs were protected with covers and the chip was rotated to fill the remaining reservoirs with the appropriate liquid and to protect with covers the remaining reservoirs. The concentration profiles after the protection of chip’s reservoirs are not susceptible to any movement of the chip (Video S1).

#### 4.2.2. Two-Substance Counter-Current Gradients Generation

40 μL of CTAB solution was added to the IC reservoir and 40 μL of first (A) active substance solution in the culture medium of maximal tested concentration was added to the inlet reservoirs of CCs. Fluids were introduced to the chambers by tilting the microdevice. All reservoirs were emptied using an automatic pipette and the fluids only remain in the chambers. The 15 μL of a second (B) active substance solution in a culture medium of the maximal tested concentration was added to the inlet reservoirs of CCs and the same volume of an indicator solution was added to the inlet reservoir of IC. The microsystem was raised at an angle of 60° to introduce the fluids into the chambers and was held in that position until all reservoirs were emptied. Then, fluids were removed from the outlet reservoirs. Afterward, again 2.0 μL of appropriate fluid was added to each reservoir (total volume of swing fluid was 4 μL). In the next step, the gradients were prepared by tilting alternately of the microsystem by 60° once by once, repeating the process 25 times. Next, the reservoirs were filled with 25 μL of fluid of the corresponding composition (test compound A or B solutions, indicator solution, or complementary liquid). After securing the reservoirs with covers the microsystem was placed in a Petri dish and placed in the incubator at 37 °C, 5% CO_2_, and 95% relative humidity. (Video S1)

### 4.3. The Media Exchange Protocol

The standard protocol of cell culture in a gradient of the active substance covers a period of 24 h. If a longer period of cell culture in contact with the active substance is required, the media in the reservoirs should be exchanged every 24 h. The media exchange procedure does not cause the change of the concentration profiles in CCs (control culture and the culture in a concentration gradient) and in IC if properly carried out. It is important that the reservoir’s cover on one side of the chip only is removed at once, and the fluids are exchanged in reservoirs on one side of the chip at once. After securing the reservoirs’ cover, the procedure can be repeated on the other side of the chip. Each time the fluids from reservoirs are removed by the pipette, and 25 μL of fresh culture media or dye solutions with appropriate (initial) concentrations of substances should be added to appropriate reservoirs. This also guarantees that the oxygen, nutrients, and metabolites are kept at the appropriate level of concentration in CCs. If necessary, this procedure can be repeated several times.

## 5. Patents

(1) Szafran Roman, Kazimierz Gąsiorowski, Katarzyna Gębczak, Benita Wiatrak. Microfluidic device for cell culture in gradient of bioactive substance, WO2018106132 (A1), 14 June 2018.
(2) Szafran Roman, Kazimierz Gąsiorowski, Katarzyna Gębczak, Benita Wiatrak. Microfluidal device for growing cell culture in a gradient of bioactive substance, PL237365B1, 06 April 2021.
(3) Szafran Roman. Method for manufacturing micro fluidized bed device for growing cell culture in a gradient of bioactive substance, PL238206B1, 26 July 2021.
(4) Szafran Roman, Kazimierz Gąsiorowski, Katarzyna Gębczak, Benita Wiatrak. Method for producing stable active substance concentration gradient in the cell culture microsystems, PL419813 (A1), 23 September 2017.

## Figures and Tables

**Table 1:** Parameters of Equation (4) estimated from the experimental data.

S_D_	A	k	X_0_	R^2^
2/34	0.011386	14.6252	0.51137	0.998
4/34	0.067666	14.7650	0.47598	0.997
8/34	0.13819	14.1625	0.47915	0.996

## Data Availability

The data presented in this study are available on request from the corresponding author.

## Supplementary Materials

The following are available online, Video S1: The procedure of two-substance counter-current gradients generation in a twelve-chamber microdevice.

## Sample Availability

Researchers wishing to collaborate on the use of a microfluidic device for their research are requested to contact the corresponding author.

## Appendix A

The visualization of stability over time of two-substance counter-current dye concentration profiles (fluorescein and methylene blue) in CC is presented in Figure A1.
